# Vocabulary Demands of Informal Spoken English Revisited: What Does It Take to Understand Movies, TV Programs, and Soap Operas?

**DOI:** 10.3389/fpsyg.2022.831684

**Published:** 2022-02-21

**Authors:** Hung Tan Ha

**Affiliations:** School of Foreign Languages, University of Economics Ho Chi Minh City (UEH), Ho Chi Minh City, Vietnam

**Keywords:** lexical coverage, BNC, COCA, TV programs, movies, soap operas

## Abstract

The article presents a methodological update on the lexical profile of informal spoken English with the emphasis on movies, television programs, and soap operas. The study analyzed Mark Davies’s mega-corpora with data containing approximately 625 million words and employed Paul Nation’s comprehensive and up-to-date British National Corpus/Corpus of Contemporary American English (BNC/COCA) wordlists. Data from the analyses showed that viewers would need a vocabulary knowledge at 3,000 and 5,000 words frequency levels to understand 95 and 98% of the words in scripted dialogs, respectively. Soap operas were found to be less lexically demanding compared to TV programs and movies. Findings are expected to fill in the methodological gaps between vocabulary assessment and vocabulary profiling research.

## Introduction

Vocabulary is the most fundamental aspect in language, and the importance of vocabulary has been constantly repeated (Nation, 2013; Webb, 2020). Together with the development of wordlists, such as the British National Corpus (BNC) lists (Nation, 2006) and the British National Corpus/Corpus of Contemporary American English (BNC/COCA) lists (Nation, 2017), researchers in the field of vocabulary studies have been continuously giving us interesting perspectives regarding our vocabulary knowledge as well as the lexical resource we would need to comprehend different text genres (Nurmukhamedov and Webb, 2019).

While vocabulary assessment and vocabulary profiling could be said to be the two fields of vocabulary studies that receive the most attention, it does seem to me that research in vocabulary testing are moving way so faster that findings in vocabulary profiling are almost left behind, causing certain gap in research methodology. For example, while vocabulary tests have long employed Nation’s (2017) up-to-date BNC/COCA wordlist as the source of test items (McLean and Kramer, 2015, 2016; McLean et al., 2015; Webb et al., 2017), many recent studies on lexical coverage of texts were still stick to the Nation’s (2006) BNC wordlist (Al-Surmi, 2014; Dang and Webb, 2014; Webb and Paribakht, 2015; Nurmukhamedov, 2017; Tegge, 2017). This led to the situation where researchers who utilized these modern vocabulary tests for their studies could not reliably relate their results to the existing findings in the field. Attempts have been made to fill in the methodological gaps (Hsu, 2018; Yang and Coxhead, 2020; Nurmukhamedov and Sharakhimov, 2021), however, they are few, and certain areas of the field, including scripted and unscripted spoken discourses, remained uncovered. As a result, research on the relationship between phonological vocabulary knowledge and listening comprehension (Cheng and Matthews, 2018; Lange and Matthews, 2020; Ha, 2021b) still had to rely on the findings of Webb and Rodgers (2009a,b), which are more than 10 years old and ripe for being updated. In response to the dire need for methodologically updated findings, the present study was conducted to revisit the vocabulary demands of informal spoken English.

## Literature Review

### Receptive Vocabulary Knowledge and Listening Comprehension

For decades, vocabulary linguists have documented a strong link between receptive vocabulary knowledge and listening comprehension (van Zeeland and Schmitt, 2013; Cheng and Matthews, 2018; Lange and Matthews, 2020; Ha, 2021b). One of the most interesting findings was the concept of *lexical demand* and *lexical coverage*. In short, lexical demand refers to the proportion of words in a text a learner need to know to adequately comprehend it. It has been generally agreed that the minimum threshold for acceptable comprehension is 95% coverage and the coverage for optimal text comprehension would be 98% (Schmitt et al., 2011; van Zeeland and Schmitt, 2013). As Hu and Nation (2000) explained, when learners knew 95% of the running word in a text, they would encounter an unfamiliar token in every 20 words, and that ratio would be reduced to 1/50 if they were to be familiar with 98% of the tokens, a huge gap for a 3% difference.

### Lexical Profile of Spoken English

The best thing about lexical coverage studies is that they are often based on word frequency lists, which would show teachers and learners the fastest route to achieve their teaching or learning targets. These wordlists use “word family” as word counting unit. A word family generally refers to a headword and all of its inflectional and derivational forms through a level 6 affix criteria (also known as WF6; Bauer and Nation, 1993; Nation, 2020). For example, the WF6 for *add* in Nation’s (2017) BNC/COCA lists includes *added, adding, addition, additional, additionality, additionally, additions, additive, additives, adds.* The WF6 have been the foundation for several aspects of vocabulary studies (Nurmukhamedov and Webb, 2019).

Past studies based on Nation (2006) British National Corpus (BNC) have formed comprehensive guidelines for English teachers and learners on what and how much to learn. For example, for informal, spoken English, learners would need to know the around 2,000–3,000 word families to achieve 95% coverage and 5,000–7,000 word families for 98% coverage (Nation, 2006; Webb and Rodgers, 2009a,b; Al-Surmi, 2014; Tegge, 2017). Academic spoken English, the type of English we would encounter in TED talks, academic seminars, and university lectures, was a little bit more lexically demanding, generally requiring a knowledge of 4,000 and 8,000 most frequent word families in the BNC word list for 95 and 98% coverage, respectively, (Coxhead and Walls, 2012; Dang and Webb, 2014; Nurmukhamedov, 2017).

### Research Gap and the Present Study

Improvement demands changes. As the English we use keeps developing every day, it is not surprising to say that the guidelines for vocabulary teaching and learning that has been built on Nation’s (2006) BNC lists would soon become obsolete, and therefore, require revisiting (Schmitt et al., 2017). In an influential paper, Schmitt et al. (2017) suggested two directions that future studies should take to replicate past lexical profile research. The first one is to increase the sample size. This statement was made based on the fact that these past studies employed relatively small corpora and their findings “now need to be checked with larger, more comprehensive corpora” (Schmitt et al., 2017, p. 217). The second suggestion Schmitt et al. (2017) put forward is the improvement in research methodology. Despite being extremely helpful and informative, Nation’s (2006) BNC word list is now 15 years old and contains primarily British English which should be “due for updating and revision” (Schmitt et al., 2017, p. 218). In an attempt to create a better version of the BNC, Paul Nation introduced the British National Corpus/Corpus of Contemporary American English (BNC/COCA) wordlist in 2012, which were later updated in 2017. The BNC/COCA is a highly regarded wordlist by researchers (Dang and Webb, 2016; Dang et al., 2020). As Schmitt et al. (2017) pressed, “Assuming the new combined BNC-COCA lists are a better indication of word frequency, then everything that has been done using the original BNC-based lists is ripe for replication using these new lists” (Schmitt et al., 2017, p. 218).

The present study was conducted in response to Schmitt et al. (2017) call and aimed at revisiting the vocabulary demands of informal spoken English. People often believe that the investigation of informal spoken English should involve real-life, conversations (Love et al., 2017). However, lexical research demonstrated that the examination on scripted English would yield similar results and be of as much help (Webb and Rodgers, 2009a,b; Al-Surmi, 2014; Davies, 2021). To date, four studies have been conducted to investigate the lexical profile of informal spoken English through soap operas (Al-Surmi, 2014), podcasts (Nurmukhamedov and Sharakhimov, 2021), TV programs (Webb and Rodgers, 2009a), and movies (Webb and Rodgers, 2009b). Tegge (2017) is not counted because song lyrics do not always reflect real-life conversations. Nurmukhamedov and Sharakhimov (2021) is very updated with their research methodology and a replication study would be unnecessary. As a result, only three of them need to be revisited in Schmitt et al. (2017) terms. Table 1 shows information regarding the sample size, the wordlist that was used as research methodology as well as the key findings of these past studies.

**Table 1:** A summary of past studies on the vocabulary demands of soap operas, TV programs, and movies.

Corpus	Word list used	Number of episodes	Number of words	Findings
Soap operas Al-surmi (2014)	BNC (Nation, 2006)	254	1,290,000	2000 WFs—95% 5,000 WFs—98%
TV programs Webb and Rodgers (2009a)	BNC (Nation, 2006)	88	264,384	3,000 WFs—95% 7,000 WFs—98%
Movies Webb and Rodgers (2009b)	BNC (Nation, 2006)	318	2,841,887	3,000 WFs—95% 6,000 WFs—98%

WF, Word Family.

By employing the most comprehensive and updated wordlist, the BNC/COCA, as well as the largest corpus of scripted English available (more details in the methodology section), the present study seeks to answer only one research question:

*Would the previous findings concerning the lexical demands of TV, Movies, and Soap Opera change as larger corpora and the BNC/COCA wordlist were applied?*

## Methodology

### Data Collection

The data analyzed in the present study were the TV (Davies, 2019a), Movies (Davies, 2019b), and SOAP (Davies, 2012) corpora, which were officially purchased and used under an academic license provided by Mark Davies.^1^ The TV, Movies, and SOAP corpora together could be said to be the largest available corpus of informal spoken English with data containing approximately 625 million tokens in total (Davies, 2021). Information regarding the three corpora is presented in Table 2.

**Table 2:** General information about the corpora (Davies, 2012, 2019a,b).

Corpus	Period	Number of episodes/scripts	Number of words
Soap operas	2001–2012	22,000	100,783,900
TV programs	1950–2018	75,000	326,201,276
Movies	1930–2018	25,000	199,479,302
Total		122,000	626,464,478

### Data Analysis

Preliminary analysis showed three major issues that had to be dealt with before the purchased corpora could be ready for any further data analysis. The first and most important thing was context-defining words. Davies’s spoken corpora are normally flooded with words that “represent the tone of style of speech” (Davies, 2021, p. 16) or gives additional information on the context, all of which are surrounded by parentheses, for example, (*Enginechuggingnoisily*), (*doorknocking*), and (*treecracking*), (*gunfire*). These words are “non-speech” words, (Davies, 2021, p. 16), and therefore, should not be included in the analysis. Another problem that needed attention was hyphenated words (*second-hand, sky-high*…) as lexical profiling software could not read them. The third issue involves words that accidentally stick together (*whatcouldpossiblybegoing, whatchancehasawomangot*…) and other typos errors, which were falsely classified by lexical profiling software as “Not in the lists.”

The parentheses that surrounded context-defining words were replaced with “<” and “>,” so that Range could identify and automatically exclude these words through the “ignore ‘< >’” function. These words accounted for 592,690, 2,577,943, and 1,689,465 tokens in the SOAP, TV, and MOVIES corpora, respectively. Hyphens in hyphenated words were then replaced by space so that the component words could be classified according to their frequency level. Finally, words that were classified as “Not in the lists” due to typos were then changed and returned to their frequency levels. These modifications were made using the mass search and replace function of Notepad++ (hotkeys: Ctrl + Shift + F).

The corpora’s lexical profile was then analyzed by Range (Heatley et al., 2002). Range is a computer program that could classify words to their frequency levels in accordance with the word lists we chose to use it with. Range were chosen for data analysis due to the researcher’s personal preference and familiarity. In fact, an analysis with AntWordProfiler 1.5.1 (Anthony, 2021) yielded near-identical results for the corpora. Therefore, such program choice should not be the cause for concern. Range can automatically identify and read contractions (cannot, do not…) and connected speech (wanna, gonna, kinda…). For instance, Range counts *cannot* as *can* and *not* and *wanna* as a family member of *want.*

The up-to-date, comprehensive, 25-level BNC/COCA wordlist (Nation, 2017) were used together with Range for the analysis. The BNC/COCA wordlist contains twenty-five 1,000-word levels which reflects current British and American English. The BNC/COCA lists are accompanied by four supplementary lists of proper nouns (*Abraham, Portuguese, Waterloo…*), marginal words (*hm, yee, phew…*), transparent compounds (*racecar, railway, sailboat…*), and acronyms (*PHD, NATO, MPHI…*; Nation, 2020).

## Results

Table 3 presents the number of words and their proportion at each frequency level in the BNC/COCA wordlist for the SOAP, TV, and Movies corpora. Proper nouns, marginal words, transparent compounds, and acronyms were treated as separate word levels.

**Table 3:** The number of tokens at each word level.

Word list	Soap operas		TV programs		Movies	
	Token	Percentage	Token	Percentage	Token	Percentage
1,000	88,429,576	83.211	274,421,665	84.737	167,842,359	85.465
2,000	2,802,518	2.637	13,285,921	4.102	7,442,263	3.790
3,000	904,801	0.851	5,264,468	1.626	2,707,299	1.379
4,000	661,411	0.622	3,287,219	1.015	1,905,709	0.970
5,000	373,582	0.352	2,262,514	0.699	1,303,088	0.664
6,000	406,273	0.382	1,438,116	0.444	804,752	0.410
7,000	148,023	0.139	894,267	0.276	513,066	0.261
8,000	325,532	0.306	790,405	0.244	480,565	0.245
9,000	133,064	0.125	630,785	0.195	357,809	0.182
10,000	101,528	0.096	405,553	0.125	212,453	0.108
11,000	121,723	0.115	371,479	0.115	200,019	0.102
12,000	39,175	0.037	296,720	0.092	162,351	0.083
13,000	31,546	0.030	214,223	0.066	138,193	0.070
14,000	16,172	0.015	158,327	0.049	87,108	0.044
15,000	13,456	0.013	125,456	0.039	72,640	0.037
16,000	10,716	0.010	94,951	0.029	51,432	0.026
17,000	14,145	0.013	86,102	0.027	49,654	0.025
18,000	5,749	0.005	59,239	0.018	33,576	0.017
19,000	7,517	0.007	55,526	0.017	32,049	0.016
20,000	22,218	0.021	47,365	0.015	25,496	0.013
21,000	11,693	0.011	32,524	0.010	18,716	0.010
22,000	1,752	0.002	26,938	0.008	16,209	0.008
23,000	3,494	0.003	24,791	0.008	15,384	0.008
24,000	6,980	0.007	14,304	0.004	10,604	0.005
25,000	1,900	0.002	14,351	0.004	9,029	0.005
Proper nouns	7,880,203	7.415	8,284,432	2.558	4,868,519	2.479
Marginal words	2,882,002	2.712	7,942,157	2.452	4,998,170	2.545
Transparent compounds	208,202	0.196	1,027,350	0.317	578,711	0.295
Acronyms	597,920	0.563	1,849,428	0.571	1,181,463	0.602
Not in the lists	108,330	0.102	445,612	0.138	269,403	0.137
Total	106,271,199	100	323,852,190	100	196,388,091	100

It is observable that around 85% of the three corpora were made up of the most frequent word families in the BNC/COCA and that nearly 90% of the corpora’s tokens were covered by the first two most frequent word families. This may be because the first two 1,000-word levels in the BNC/COCA lists primarily contain words taken from spoken corpora (Nation, 2020). The proportion of tokens showed a gradual decrease as the word frequency went down, and after the 5,000 level, the figures dropped below 1% for all the corpora, signaling the importance of high-frequency words.

Another detail that deserves attention is the proportion of proper nouns in the three corpora, which were relatively considerable, especially for the SOAP corpus. Proper nouns normally do not cause significant difficulties for reading comprehension since they can be easily recognized with the first-letter capitalization. However, concerns have been raised on the effect of proper nouns on listening comprehension (Kobeleva, 2012; Klassen, 2021). In general, auxiliary words including proper nouns (PN), marginal words (MW), transparent compounds (TC), and acronyms accounted for approximately 11% for the SOAP corpus and nearly 6% for the TV and Movies corpora.

The cumulative coverage at each word frequency level is illustrated in Table 4. At this stage, two assumptions were put forward, the first one supposes that learners did not know and could not recognize PNs, MWs, TCs, and acronyms, and the second one assumes that learners knew or could easily recognize these words.

**Table 4:** Cumulative coverage with and without proper nouns, marginal words, transparent compounds, and acronyms.

Word List	Soap operas		TV programs		Movies	
	Without	With	Without	With	Without	With
1,000	83.211	94.097	84.737	90.635	85.465	91.385
2,000	85.848	96.734	88.839	94.738	89.254	95.175
3,000	86.700	97.585	90.465	96.364	90.633	96.553
4,000	87.322	98.208	91.480	97.379	91.603	97.523
5,000	87.674	98.559	92.178	98.077	92.267	98.187
6,000	88.056	98.942	92.622	98.521	92.676	98.597
7,000	88.195	99.081	92.899	98.797	92.938	98.858
8,000	88.502	99.387	93.143	99.041	93.182	99.103
9,000	88.627	99.512	93.337	99.236	93.365	99.285
10,000	88.722	99.608	93.463	99.361	93.473	99.393
11,000	88.837	99.723	93.577	99.476	93.575	99.495
12,000	88.874	99.759	93.669	99.568	93.657	99.578
13,000	88.903	99.789	93.735	99.634	93.728	99.648
14,000	88.919	99.804	93.784	99.683	93.772	99.692
15,000	88.931	99.817	93.823	99.722	93.809	99.729
16,000	88.941	99.827	93.852	99.751	93.835	99.756
17,000	88.955	99.840	93.879	99.777	93.860	99.781
18,000	88.960	99.846	93.897	99.796	93.878	99.798
19,000	88.967	99.853	93.914	99.813	93.894	99.814
20,000	88.988	99.874	93.929	99.828	93.907	99.827
21,000	88.999	99.885	93.939	99.838	93.916	99.837
22,000	89.001	99.886	93.947	99.846	93.925	99.845
23,000	89.004	99.890	93.955	99.854	93.932	99.853
24,000	89.011	99.896	93.959	99.858	93.938	99.858
25,000	89.012	99.898	93.964	99.862	93.942	99.863
Total	106,271,199		323,852,190		196,388,091	

It is obvious that without the knowledge of PN, MW, TC, and acronyms, it is impossible for viewers to achieve the minimum coverage threshold for comprehension, which is indeed worrying. However, when PN, MW, TC, and acronyms were assumed to be known, then we can once again see the optimistic scenario depicted in Webb and Rodgers (2009a,b), Tegge (2017), and Nurmukhamedov and Sharakhimov (2021). Generally, it only took 2,000–3,000 most frequent word families in the Nation’s (2017) BNC/COCA lists to cover 95% of the tokens in the corpora, and the vocabulary knowledge of the most frequent 4,000–5,000 word families was all required to reach the optimal threshold for comprehension.

Certain differences can be observed between the corpora. To be more specific, soap operas demanded the least lexical knowledge for 95% (2,000 WFs) and 98% (4,000 WFs) coverage compared to the other two. TV programs and movies share similar lexical demands when it comes to the 98% threshold. However, data from the analysis showed that the Movies corpus managed to reach the 95% coverage at the 2,000 level, while the TV programs would require a word knowledge at 3,000 level for 95% coverage. Still, it worth noting that the actual difference was really thin, approximately 0.2%, and had the tendency to become smaller as it moved down the word levels. Therefore, it is safe to state that movies and TV programs shared relatively similar vocabulary demands.

## Discussion

The paper revisited research on the vocabulary profiles of soap operas (Al-Surmi, 2014), TV programs, and movies (Webb and Rodgers, 2009a,b) to see if changes in sample size and research methodology would result in changes in findings. Data from the analyses showed that if the BNC/COCA wordlist were to be used as an indicator of word frequency, then the lexical demands of the researched text genres would be generally reduced. To be more specific, if learners were to base their vocabulary learning on the BNC/COCA lists, they would only need to learn 4,000 (instead of 5,000) and 5,000 (instead of 6,000 and 7,000) word families to understand 98% of the words in soap operas and movies and TV programs, respectively. It should be noted that the 1,000–2,000 word families difference could be translated into 2–4 years of English learning or even more (Webb and Chang, 2012; Ozturk, 2016).

However, the so-called “reduced lexical demands” are, in actual practice, the additional effect of the four supportive lists (PN, MW, TC, and Acronym). If we were to compare the cumulative coverage at the 3,000 and 5,000 levels, we could easily find similar figures between BNC/COCA-based and BNC-based studies. And if we were to go even further and add the proportion of the four supportive lists (PN, MW, TC, and Acronym) of this study to the cumulative coverage figures at any word level in Webb and Rodgers (2009a,b) and Al-Surmi (2014), then the same reduced lexical coverage (or even better) could be recorded. Still, this does not mean that we could simply use the BNC lists together with the four additional lists in the BNC/COCA lists. Nation (2020) introduced the BNC/COCA wordlist with clear rationales which have been proven by other researchers (Dang and Webb, 2016; Dang et al., 2020), which means that these lists were designed to work together and scholars are not encouraged to apply questionable practices to avoid unnecessary problems.

The shift in lexical profiling studies from the BNC (Nation, 2006) to the BNC/COCA (Nation, 2017) could be said to be inevitable as it harmonizes different aspects of vocabulary research. Since most vocabulary tests now utilize the BNC/COCA as the source for test items (McLean and Kramer, 2015; McLean et al., 2015; Webb et al., 2017; Ha, 2021a), it would be somehow methodologically inconsistent to relate students’ results on vocabulary test that is based on the BNC/COCA wordlist to findings of lexical studies that employed the BNC lists. Together with the development of phonological vocabulary tests (McLean et al., 2015; Ha, 2021a), teachers and vocabulary linguists can now make a reliable connection between the aural vocabulary knowledge of their students and what they can possibly understand in the real world.

The study’s findings were also in line with Nurmukhamedov and Sharakhimov (2021) which employed Nation’s (2017) BNC/COCA lists to investigate the lexical profile of English podcasts. Nurmukhamedov and Sharakhimov (2021) found that a vocabulary knowledge at the 3,000 and 5,000 levels would cover 96.75 and 98.26% of the words in English podcasts, correspondingly. These results generally suggested that learning the 5,000 most frequent word families in the BNC/COCA wordlist, an attainable learning goal for English learners in most contexts, could help learners achieve unsupported listening comprehension for informal spoken English. This claim is supported by van Zeeland and Schmitt’s (2013) study which proved that knowing 98% of the running words in a listening text would result in very high degree of listening comprehension.

The study recorded considerable proportions of PN, MW, TC, and acronyms, which aligned well with Nurmukhamedov and Sharakhimov (2021). Vocabulary linguists tend to make the assumption that these words could be easily and recognized by learners (Nation, 2006; Webb and Rodgers, 2009a,b; Nurmukhamedov and Sharakhimov, 2021). Although these assumptions could be acceptable for reading, concerns have been raised about whether it is appropriate to assume the same thing to listening (Kobeleva, 2012; Klassen, 2021). It is true that without the support of orthographic form, proper nouns or even acronyms are difficult to distinguish, and for listening-only formats like podcasts, such concerns are in evidence. However, television programs, movies, and soap operas, which are strongly supported by visualization, are different from podcasts. These non-verbal clues, such as facial expression, body gestures, and lips movements, are considered be of significant support to the processing of aural input (Harris, 2003). Such visual clues may help viewers recognize and understand PN, MW, TC, and acronyms.

## Conclusion

This research’s findings offer updates on the lexical profiles of informal spoken English by employing up-to-date research methodology and large sample size. In general, it is evident that the BNC/COCA wordlist (Nation, 2017) would give English learners and teachers a shorter route to their intended goals. This BNC/COCA-based update also connected research findings on vocabulary profiling and vocabulary assessment, which have been in conflict for several years due to incompatible methodologies.

Despite being informative, this brief research report bears certain limitations. First, the paper revisited the findings of several studies at once, which would give readers a broad overview of the new findings and how these findings related to each other. Therefore, it was not possible for the researcher to go deeper and explore the variation in lexical coverage among texts. Future research should take a deeper look into each corpus in isolation and examine the variation in lexical demands of each text genre. Secondly, although the study showed the number of word families learners would need to achieve 95 and 98% coverage in informal spoken English. It cannot guarantee that learners would successfully comprehend a text should these requirements be satisfied. Graham (2006) showed that people may understand every single word in a text and still fail to get the general meaning, which could be due to other factors in the learners’ language proficiency and metacognitive awareness. As a result, researchers are encouraged to investigate these issues, which could possibly be done by replicating van Zeeland and Schmitt’s (2013) research methodology.

## Data Availability Statement

The data analyzed in this study is subject to the following licenses/restrictions: The corpora that support the findings of this study are available from Mark Davies. Restrictions apply to the availability of these corpora, which were used under academic license for this study. Data are available from https://www.english-corpora.org/ with the permission of Mark Davies. Requests to access these datasets should be directed to mark.davies@corpusdata.org.

## Author Contributions

The author confirms sole responsibility for the following: study conception and design, data collection, analysis and interpretation of results, and manuscript preparation.

## Conflict of Interest

The author declares that the research was conducted in the absence of any commercial or financial relationships that could be construed as a potential conflict of interest.

## Publisher’s Note

All claims expressed in this article are solely those of the authors and do not necessarily represent those of their affiliated organizations, or those of the publisher, the editors and the reviewers. Any product that may be evaluated in this article, or claim that may be made by its manufacturer, is not guaranteed or endorsed by the publisher.

## Footnotes

^1^ https://www.english-corpora.org/

## Abbreviations

BNC: British National Corpus
COCA: Corpus of Contemporary American English
ESL: English as a second language
MW: Marginal words
NOW: News on the web
PN: Proper nouns
TC: Transparent compounds
